# Truck Cargo Load Change Detection: Depth Map Normalization and 1D Projection Matching

**DOI:** 10.3390/s26185866

**Published:** 2026-09-16

**Authors:** Yongju Park, Changil Kim, Yuntae Kim, Jinuk Park, Byeong-Kwon Ju

**Affiliations:** 1 Display and Nanosensor Laboratory, Department of Micro/Nano Systems, Korea University, Seoul 02841, Republic of Korea; suede82@korea.ac.kr or suede8247@keti.re.kr (Y.P.); yt96@korea.ac.kr or kimyoontae@keti.re.kr (Y.K.); 2 Smart Network Research Center, Korea Electronics Technology Institute, Seongnam-si 13449, Republic of Korea; mykwor2468@keti.re.kr (C.K.); jinuk.park@keti.re.kr (J.P.)

**Keywords:** cargo load change detection, depth map normalization, 1D projection matching, image alignment, stereo vision, driver safety

## Abstract

Cargo-fall accidents caused by improper cargo loading have frequently occurred on highways in recent years, posing a growing threat to driver safety. Because enforcement typically relies on fixed inspection stations, some drivers illegally alter their cargo loads between checkpoints to evade overload inspections, and such changes are rarely secured properly, further increasing the risk of cargo-fall accidents. Current cargo inspection systems, however, are largely limited to labor-intensive manual checks or weight-based sensors that cannot distinguish between items of comparable weight. This paper presents an approach that detects cargo load changes on trucks by comparing depth maps acquired through a stereo vision system across consecutive passes. The proposed approach normalizes the raw depth map to correct for perspective distortion and aligns the current and previous cargo-bed regions through one-dimensional projection matching, enabling accurate comparison independent of cargo color or texture. We validate the approach through field experiments at SMTB under two test scenarios, one with uniform cargo items and one with diverse, irregularly placed items, and benchmark it against four widely used alignment algorithms: SIFT, ORB, AKAZE, and XFeat. The proposed algorithm reaches a combined detection rate of 91.49% (43/47), well above every conventional algorithm, at a processing speed nearly on par with ORB, the fastest of the compared methods. Notably, all four conventional algorithms consistently miss the same subset of events involving a tarpaulin, whose color and texture closely resemble the cargo bed, whereas the depth-based approach detects these events without difficulty. These findings offer practical guidance for building more reliable, real-world cargo load change detection systems.

## 1. Introduction

Owing to the increasing volume of road freight transportation, cargo-fall accidents involving trucks have frequently occurred on highways in recent years, posing a growing threat to driver safety. According to statistics from the Ministry of Land, Infrastructure and Transport and the Korea Expressway Corporation, a total of 238 cargo-fall-related accidents occurred during the five-year period since 2020, resulting in 27 casualties. Such accidents, caused by improper cargo loading, can lead to severe consequences, including road damage and chain-reaction collisions, which often escalate into fatal traffic accidents. In particular, the occurrence of cargo falls is closely related to the loading conditions of cargo trucks.

To evade overload inspections, some drivers illegally alter their cargo loads between checkpoints [1]. Because enforcement typically relies on fixed inspection stations, drivers can adjust their payload while traveling between two checkpoints to circumvent detection. Crucially, this practice increases the risk of cargo-fall accidents, as items added or removed under time constraints are rarely secured properly. Consequently, an automated system capable of detecting such unauthorized cargo changes between checkpoints is required.

However, current cargo inspection systems have two major limitations. The first limitation is that existing cargo inspection practices rely primarily on visual inspection by human inspectors. This approach requires substantial manpower and time, remaining susceptible to errors arising from subjective judgment. Secondly, another critical limitation concerns automated weight-based systems, such as weigh-in-motion (WIM) sensors, which measure only the total weight of the cargo [2,3]. As a result, these systems fail to detect load changes in which an item is replaced with another of similar weight, as the net weight remains unchanged. Therefore, a system capable of directly capturing and comparing the actual loading state of cargo, rather than relying solely on total weight, is required to reliably detect such load changes.

This requirement leads us to employ a stereo vision system installed above the road to photograph trucks as they pass. Because the same truck may pass at slightly different positions and angles on each occasion, a direct comparison of two images captured at different passes is prone to errors. Accordingly, accurately aligning the images captured at different passes is essential to reliably distinguish genuine load changes from such positional discrepancies.

Image alignment techniques have been studied to compensate for such positional discrepancies between images captured at different times [4]. Conventional approaches are largely based on hand-crafted feature-based methods, including SIFT, ORB, and AKAZE [5,6], which detect and match distinctive keypoints through local color or intensity gradients. Recently, a learning-based method, XFeat [7], has been proposed to extract local features using a lightweight convolutional neural network. However, because both approaches operate on two-dimensional color information, their matching performance is dependent on the color and texture of the captured objects, which can degrade alignment accuracy [8].

In contrast, depth information obtained from stereo vision represents the three-dimensional geometry of a scene rather than its color, and is therefore largely independent of the color and texture of the captured cargo. Stereo vision estimates the distance to an object from the disparity between corresponding points in the left and right images [9]. Nevertheless, applying stereo vision to cargo load change detection still involves two additional issues. First, raw depth maps generated by stereo vision include perspective distortion, causing the same physical length to map to different pixel lengths depending on the distance from the camera. Second, the continuous alignment and comparison of raw three-dimensional depth data across consecutive passes incurs a high computational cost, which hinders real-time processing [10].

To address these issues, we propose a stereo vision-based cargo load change detection method for trucks. We perform the three-dimensional cargo comparison through a combination of depth map normalization and one-dimensional projection matching. Specifically, we normalize the raw depth map based on the representative height of each row to mitigate the effect of perspective distortion. Building on this normalized representation, we then align the current and previous cargo-bed regions by projecting edge information along the X- and Y-axes into one-dimensional arrays and comparing them, which substantially reduces the computational cost compared with a pixel-wise two-dimensional search. Through this approach, we aim to detect cargo load changes both accurately and in real time.

The main contributions of this study are as follows:

- We propose a stereo vision-based cargo load change detection method for trucks, which combines depth map normalization and one-dimensional projection matching to align cargo-bed images captured at different passes accurately and in real time.
- We evaluate the proposed method through field experiments, in terms of both detection performance across two test scenarios with different cargo item types and placement conditions, and comparison with conventional alignment algorithms, including SIFT, ORB, AKAZE, and XFeat.
- We identify recurring detection failure patterns from the experimental results and provide insightful analyses to facilitate future research on cargo load change detection.

The remainder of this paper is organized as follows. Section 2 briefly reviews related work on image alignment techniques. In Section 3, we introduce the proposed method, including depth map normalization and one-dimensional projection matching. Section 4 describes the experimental setup, including implementation details, test scenarios, and evaluation metrics. In Section 5 and Section 6, we present the experimental results and provide a detailed discussion of these results. Finally, Section 7 provides a conclusion and future works.

## 2. Related Work

Image alignment plays a critical role in cargo load change detection, as a truck may pass at slightly different positions and angles on each occasion. Without accurate alignment, such positional discrepancies can be misinterpreted as actual load changes, making precise alignment a prerequisite for accurate detection. To address this challenge, various image alignment methods have been studied, ranging from hand-crafted feature-based approaches to more recent deep learning-based approaches [11,12,13].

The Scale-Invariant Feature Transform (SIFT) [14] was among the earliest algorithms to achieve keypoint detection and description that remain robust to changes in scale and rotation, laying the foundation for feature-based image alignment. SIFT constructs a scale-space representation using the Difference of Gaussian and describes each keypoint with a high-dimensional gradient histogram, achieving high matching accuracy across diverse viewing conditions [15]. However, this extensive gradient-based description imposes a substantial computational burden, which limits its suitability for real-time processing.

To address this computational burden, Oriented FAST and Rotated BRIEF (ORB) was proposed as an efficient alternative to SIFT [16,17]. ORB combines the FAST corner detector with the binary BRIEF descriptor, substantially reducing both detection and matching costs while incorporating orientation compensation to preserve rotation invariance. Although this design enables considerably faster processing, the resulting binary descriptor conveys less-detailed information than gradient-based descriptors, which can reduce matching robustness under substantial scale variation.

While SIFT and ORB both rely on a linear scale space, KAZE [18] was introduced to construct a nonlinear scale space that suppresses noise while preserving object boundaries, offering more detailed descriptions than linear scale-space methods. However, computing this nonlinear scale space incurred a substantially higher computational cost. Accelerated-KAZE (AKAZE) [19] was subsequently developed to reduce this cost through the Fast Explicit Diffusion framework, retaining much of the noise robustness of KAZE at a considerably lower computational cost [20]. However, despite these differences in detection speed and descriptor design, all three methods commonly identify and match keypoints based on local intensity or gradient patterns within the image, and their matching performance deteriorates in scenes with insufficient color contrast or texture.

More recently, XFeat [7] was proposed to depart from hand-crafted detection and description by learning discriminative local features directly from data using a lightweight convolutional neural network, achieving greater robustness to illumination and viewpoint changes than earlier methods [21]. Nevertheless, because XFeat also operates on two-dimensional color information, it remains subject to the same dependency on the color and texture of the captured objects observed in hand-crafted methods.

Beyond these two-dimensional alignment methods, several recent studies have investigated alternative sensing modalities for cargo- or load-related recognition tasks. A stereo camera combined with two LiDAR sensors has been used to determine the three-dimensional position of a dump truck’s cargo box and analyze its loading space, projecting dense depth data onto the rear plane of the cargo box to estimate its initial pose [22]. In addition, a similar optical setup mounted on a load–haul–dump machine has also been employed to characterize a muck pile’s location and overall shape using a topological algorithm [23].

Unlike the color- and texture-dependent methods described earlier, depth information represents the three-dimensional geometry of a scene rather than its color, making it less dependent on the color and texture of the captured cargo. These studies, however, address the detection of cargo presence, position, or shape at a single point in time, rather than changes across separate passes of the same vehicle. Motivated by this limitation, we develop a stereo vision-based alignment method that normalizes the raw depth map and matches the current and previous cargo-bed regions through one-dimensional edge projection, which we describe in detail in Section 3.

## 3. Materials and Methods

### 3.1. Method Overview

Our goal is to detect cargo load changes between two passes of the same truck by comparing the depth maps captured during each pass. For ease of description, we first describe the overall pipeline of the proposed method, and the following subsections then present depth map normalization, one-dimensional projection matching, and load change detection in detail.

**Figure 1:**
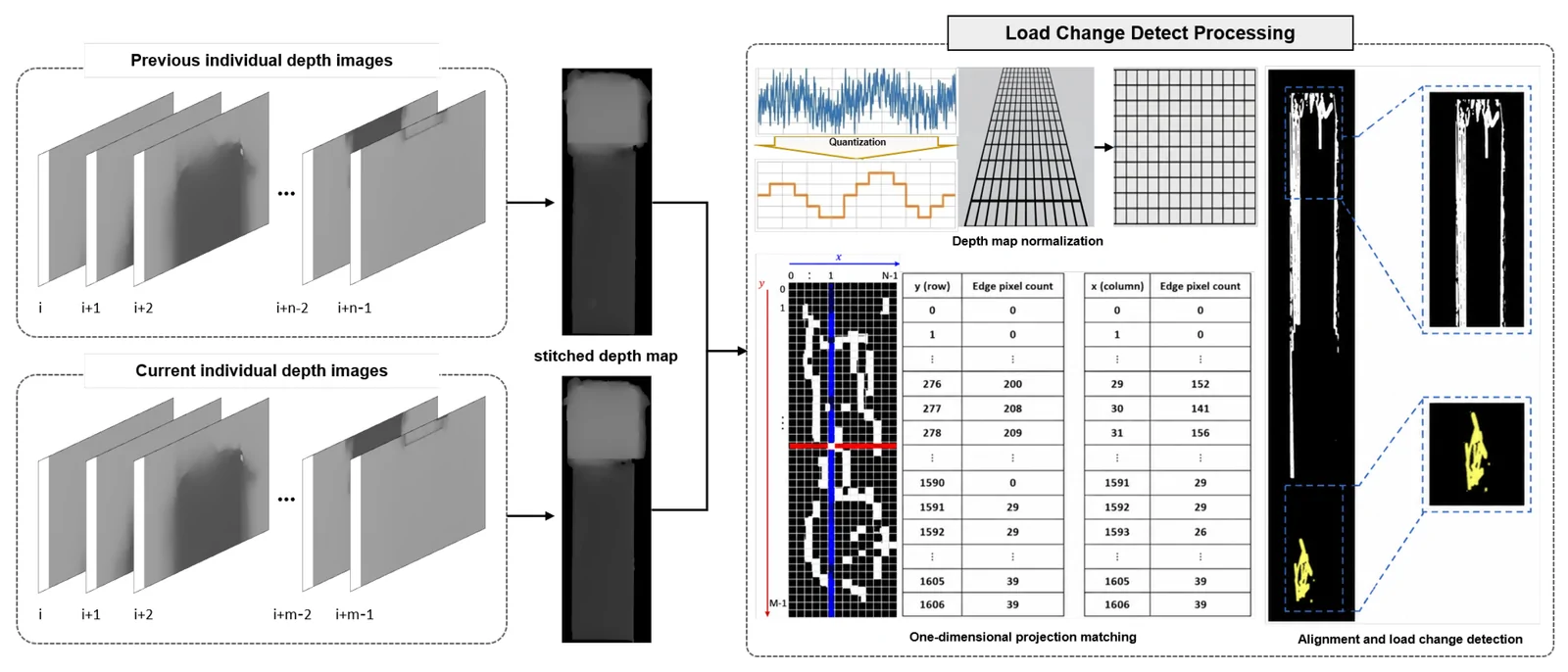
Overall processing pipeline of the proposed method, showing depth map stitching, depth map normalization, one-dimensional projection matching, and load change detection.

Figure 1 shows the overall processing pipeline of the proposed method, which consists of depth map normalization, one-dimensional projection matching, and load change detection. Once a truck has passed the stereo vision system, a sequence of individual depth images is stitched into a single depth map spanning the entire length of the truck [24,25]. This stitching process has been developed in our prior work and is not detailed in this study. It is performed separately for the previous and current passes, producing the stitched depth maps that serve as the input to the pipeline.

Each stitched depth map is first normalized based on the representative height of each row, converting it into a normalized representation that mitigates the effect of perspective distortion [26]. Using these normalized representations, the current and previous cargo-bed regions are aligned through one-dimensional projection matching, in which edge information extracted from the cargo-bed region is projected along the X- and Y-axes into one-dimensional arrays.

After alignment, the current and previous depth maps are compared to extract candidate regions of load change, and these candidates are validated to exclude regions that do not correspond to an actual load change. The validated regions are then transformed back to the coordinate space of the original depth map to produce the final detection result.

### 3.2. Depth Map Normalization

Due to the camera’s perspective projection, the raw depth map inevitably contains perspective distortion, in which the same physical length corresponds to different pixel lengths depending on the distance from the camera [27,28,29]. We conduct depth map normalization to correct this distortion, converting the raw depth map into a normalized representation in which a fixed pixel distance corresponds to a fixed real-world distance of 1 mm throughout the depth map.

To this end, we first compute a representative height value for each row of the raw depth map. The representative height H(y) for row *y* is defined as the average height value among the pixels whose quantized height corresponds to the most frequently occurring level within that row, as shown in Equation (1).

(1) $$H\left(y\right)=Q\left(\mathrm{mean}\left(\{h\left(x,y\right):Q\left(h\left(x,y\right)\right)=v^{*}\}\right)\right),\;v^{*}=\arg\max_{v}\left|\{x:Q(h(x,y))=v\}\right|$$

where h(x,y) is the raw height value at pixel (x,y), *Q* is a function that quantizes a height value into one of a set of discrete height levels, *v* denotes one such quantized height level, and $v^{*}$ is the level containing the largest number of pixels within row *y*. This quantization also suppresses the influence of noisy pixels, which would otherwise degrade the quality of the depth map when it is subsequently resized [30]. Consecutive rows sharing the same representative height are then grouped into a single segment for the subsequent resizing step.

Because the mapping between real-world distance and pixel distance varies with the distance from the camera, we define MMPP(H) as follows:

(2) $$\mathrm{MMPP}\left(H\right)=\frac{\mathrm{actual}\;\mathrm{physical}\;\mathrm{size}(H)\;[\mathrm{mm}]}{1\;\mathrm{pixel}}$$

where MMPP(H) denotes the real-world length, in mm, corresponding to one pixel at height *H*. The values of MMPP are precomputed for each height level and stored in a lookup table. For a segment with representative height H(y) spanning *n* original rows, the target segment length *s*, in pixels, is then given as follows:

(3) $$s=\mathrm{round}\left(\mathrm{MMPP}(H(y))\times n\right)$$

**Figure 2:**
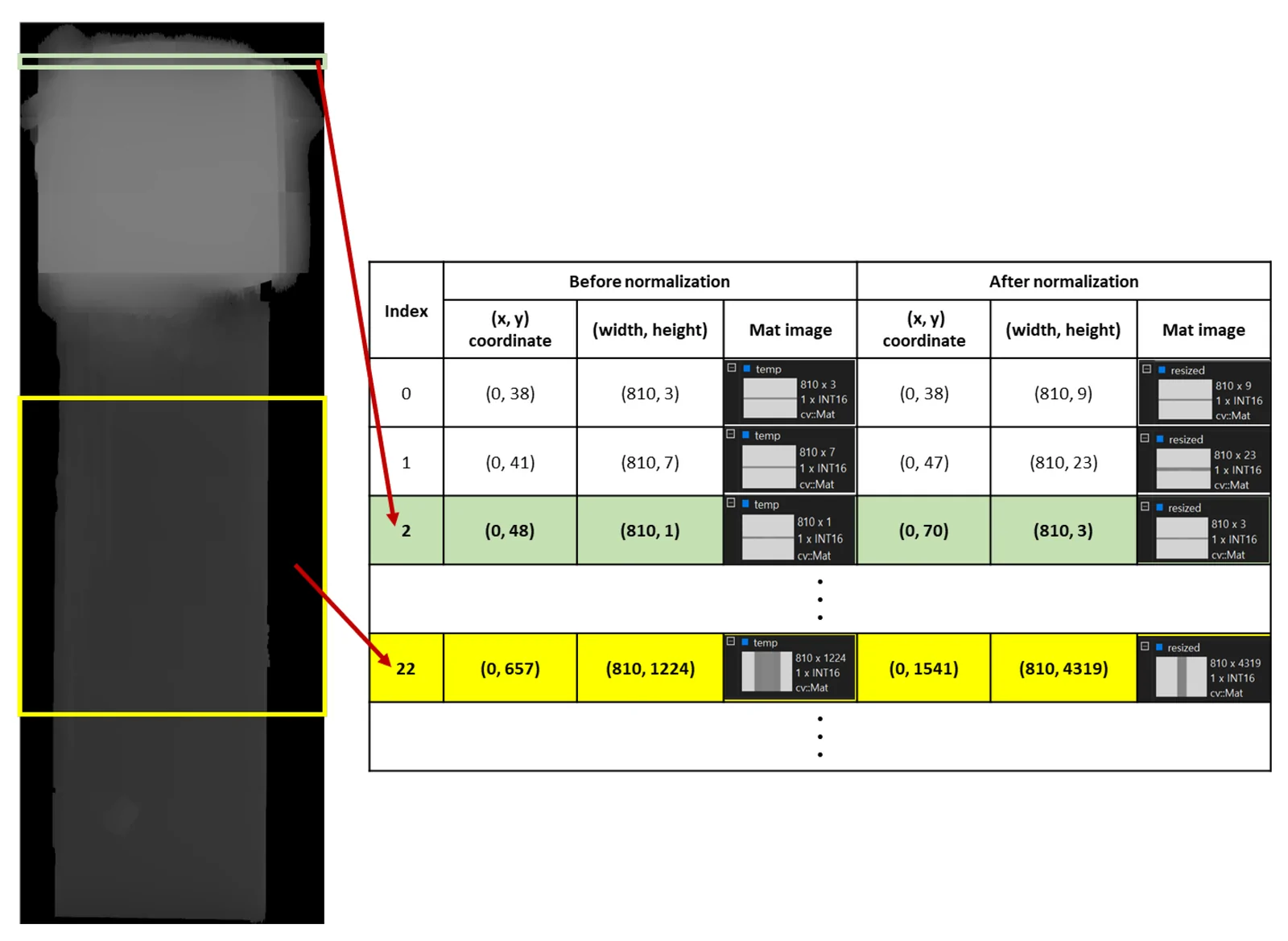
Example of segment-wise resizing during depth map normalization. The segments at index 2 and index 22 are marked in green and yellow, respectively.

Each segment of the raw depth map is then resized to a length of *s* pixels, and the resized segments are stacked in their original order to produce the normalized depth map, in which every pixel corresponds to a fixed real-world distance of 1 mm. Figure 2 presents this process using two example segments extracted from the same depth map. The segment at index 2, a thin segment spanning a single original row, is stretched to 3 pixels, whereas the segment at index 22, spanning 1224 original rows, is stretched to 4319 pixels. This difference reflects the values obtained from MMPP.

### 3.3. One-Dimensional Projection Matching

The cargo-bed region used for edge extraction and projection matching excludes the vehicle head, such as the cab of a truck, because this region cannot carry cargo and would otherwise interfere with the matching process.

**Figure 3:**
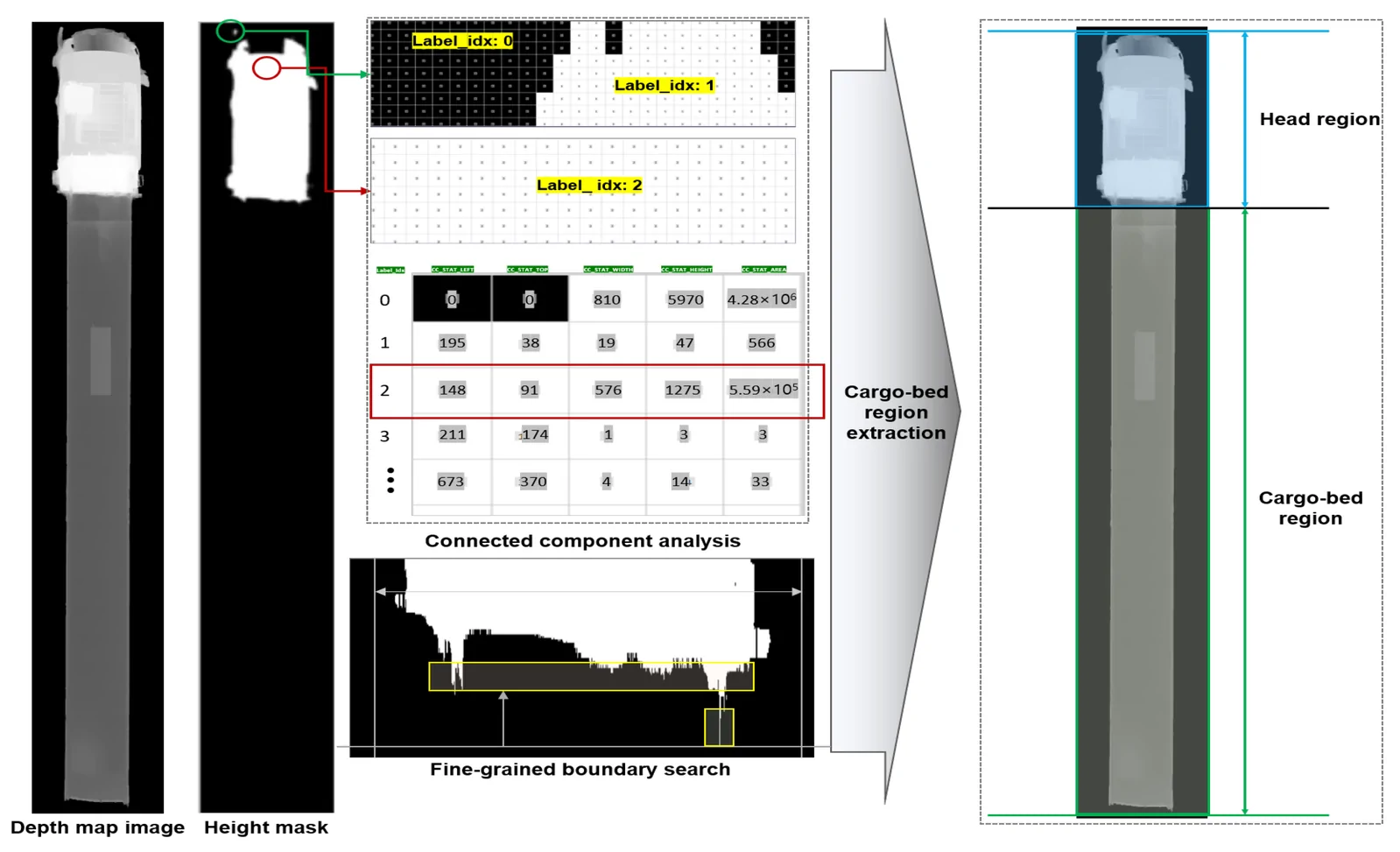
Extraction of the cargo-bed region from a depth image.

Figure 3 shows how this region is extracted. We detect the vehicle head by masking the regions of the depth map whose height exceeds a threshold, since the head is structurally taller than the cargo bed. Within this mask, the topmost connected region [31,32] that satisfies a minimum width and length is identified as the head candidate. Within this candidate region, we locate the boundary between the head and the cargo bed as the row nearest the candidate’s lower edge at which the head spans a sufficient width without any gap. The cargo-bed region is defined as the portion of the depth map beyond this boundary.

**Figure 4:**
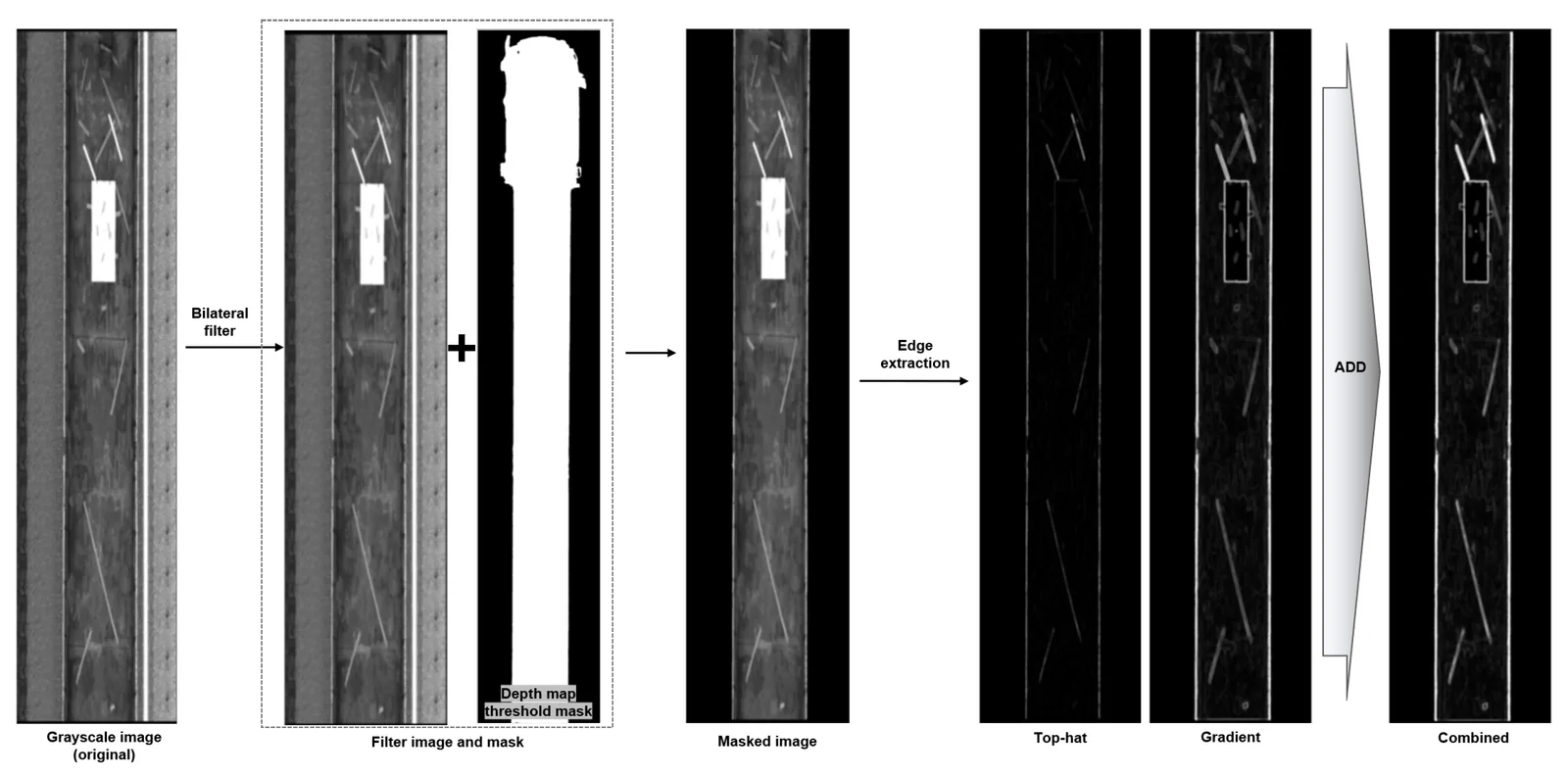
Preprocessing and edge extraction from the grayscale image of the cargo-bed region. The white box indicates a cargo item present in the example image.

We extract the edges of this cargo-bed region from a grayscale image. Figure 4 shows the preprocessing of the grayscale image for edge extraction. The grayscale image is first smoothed using a bilateral filter [33], which reduces noise while preserving edges, and then combined with a mask retaining only the region above a height threshold, yielding the preprocessed image.

To extract fine texture and boundary features from this preprocessed image, we apply the top-hat [34,35] and gradient [36] operations, both of which are based on the two fundamental morphological operations, dilation and erosion. Dilation expands the boundary of bright regions in an image, filling small gaps, whereas erosion shrinks the boundary of bright regions, removing small protrusions and noise. Combining these two operations yields opening, defined as erosion followed by dilation, and closing, defined as dilation followed by erosion.

The top-hat operation extracts small features brighter than their surroundings by subtracting the opening of the image from the original image, as follows:

(4) $$I_{top\text{-}hat}=I_{original}-I_{opening}$$

where $I_{opening}$ is obtained by applying erosion followed by dilation to $I_{original}$. The morphological gradient extracts the outline of objects by subtracting the eroded image from the dilated image, defined as:

(5) $$I_{gradient}=I_{dilation}-I_{erosion}$$

We add the outputs of $I_{top\text{-}hat}$ and $I_{gradient}$ to combine the enhanced bright features and object boundaries into a single image [37,38], shown as the combined result in Figure 4. Pixels with low intensity in this combined image are then removed using an adaptive threshold [39,40], and the remaining pixels form the final edge map $I_{edge}$.

We propose projecting this final edge map along the X- and Y-axes into two one-dimensional arrays to align the current and previous cargo-bed regions, as follows:

(6) $$e\left(x\right)=\sum_{y}I_{edge}\left(x,y\right),\;e\left(y\right)=\sum_{x}I_{edge}\left(x,y\right)$$

where e(x) and e(y) denote the number of edge pixels in column *x* and row *y*, respectively. We obtain $e_{curr}\left(x\right)$, $e_{curr}\left(y\right)$, $e_{prev}\left(x\right)$, and $e_{prev}\left(y\right)$ by applying this projection to the current and previous edge maps. Since the current and previous cargo-bed regions may differ in size, we determine the shift that minimizes the difference between their edge-count arrays [41]:

(7) $$\Delta x^{*}=\arg\min_{\Delta x}\sum_{x}\left|e_{curr}\left(x\right)-e_{prev}\left(x-\Delta x\right)\right|$$

(8) $$\Delta y^{*}=\arg\min_{\Delta y}\sum_{y}\left|e_{curr}\left(y\right)-e_{prev}\left(y-\Delta y\right)\right|$$

where all four functions are defined as zero outside their respective ranges. Although the resulting shift $\left(\Delta x^{*},\Delta y^{*}\right)$ is two-dimensional, $\Delta x^{*}$ and $\Delta y^{*}$ are determined independently by comparing one-dimensional signals along each axis, rather than jointly searching over the two-dimensional depth map; this substantially reduces the computational cost.

### 3.4. Alignment and Load Change Detection

The shift $\left(\Delta x^{*},\Delta y^{*}\right)$ obtained in Section 3.3 is applied to align the current and previous cargo-bed regions, whichever region requires shifting relative to the other, so that their overlapping areas correspond to the same physical location on the cargo bed.

After alignment, we resize the current and previous depth maps to a common width and height, since a pixel-wise comparison between the two depth maps requires them to be the same size. We then compute the height difference at each pixel in both directions, as follows:

(9) $$D_{cp}\left(x,y\right)=\max\left(H_{curr}\left(x,y\right)-H_{prev}\left(x,y\right),\;0\right),\;D_{pc}\left(x,y\right)=\max\left(H_{prev}\left(x,y\right)-H_{curr}\left(x,y\right),\;0\right)$$

where $H_{curr}$ and $H_{prev}$ denote the height values of the aligned current and previous depth maps, and $D_{cp}$ and $D_{pc}$ denote the resulting directional difference maps, computed only for pixels where both $H_{curr}$ and $H_{prev}$ exceed a minimum height above the ground. $D_{cp}$ represents candidate additions, and $D_{pc}$ represents candidate removals.

**Figure 5:**
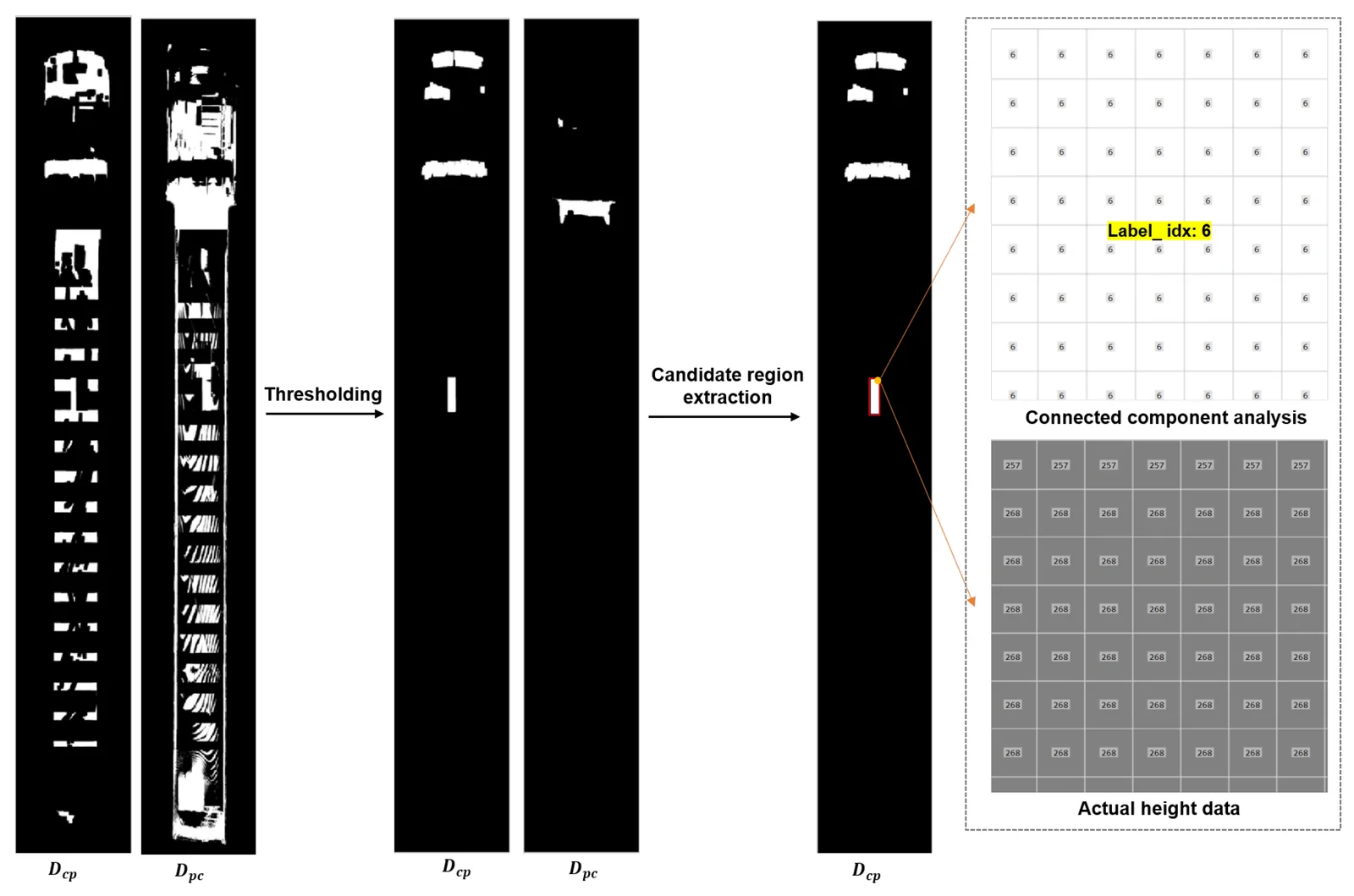
Candidate region extraction and final height-based verification process for $D_{cp}$ and $D_{pc}$.

We extract candidate regions of load change by thresholding $D_{cp}$ and $D_{pc}$ at a minimum height. Morphological opening, erosion followed by dilation, is then applied to each thresholded map to remove noise smaller than a minimum size while preserving larger candidate regions [42]. Figure 5 shows this process using an example of $D_{cp}$ and $D_{pc}$ obtained from one pass, together with the resulting candidate region extracted from $D_{cp}$.

We identify connected regions within each candidate map and validate each region against a set of criteria, including minimum and maximum width, minimum length, minimum area, and a minimum ratio of area to bounding-box area, which excludes sparse and scattered regions unlikely to correspond to a solid object. Candidate regions located within the vehicle head region or beyond the end of the cargo bed are also excluded.

For each candidate region that satisfies these criteria, we compute its average actual height and confirm it as an instance of load change when this value exceeds a minimum height. Regions derived from $D_{cp}$ are labeled as additions, and those derived from $D_{pc}$ are labeled as removals, producing the final detection result.

## 4. Experimental Setup

### 4.1. Implementation Details

**Figure 6:**
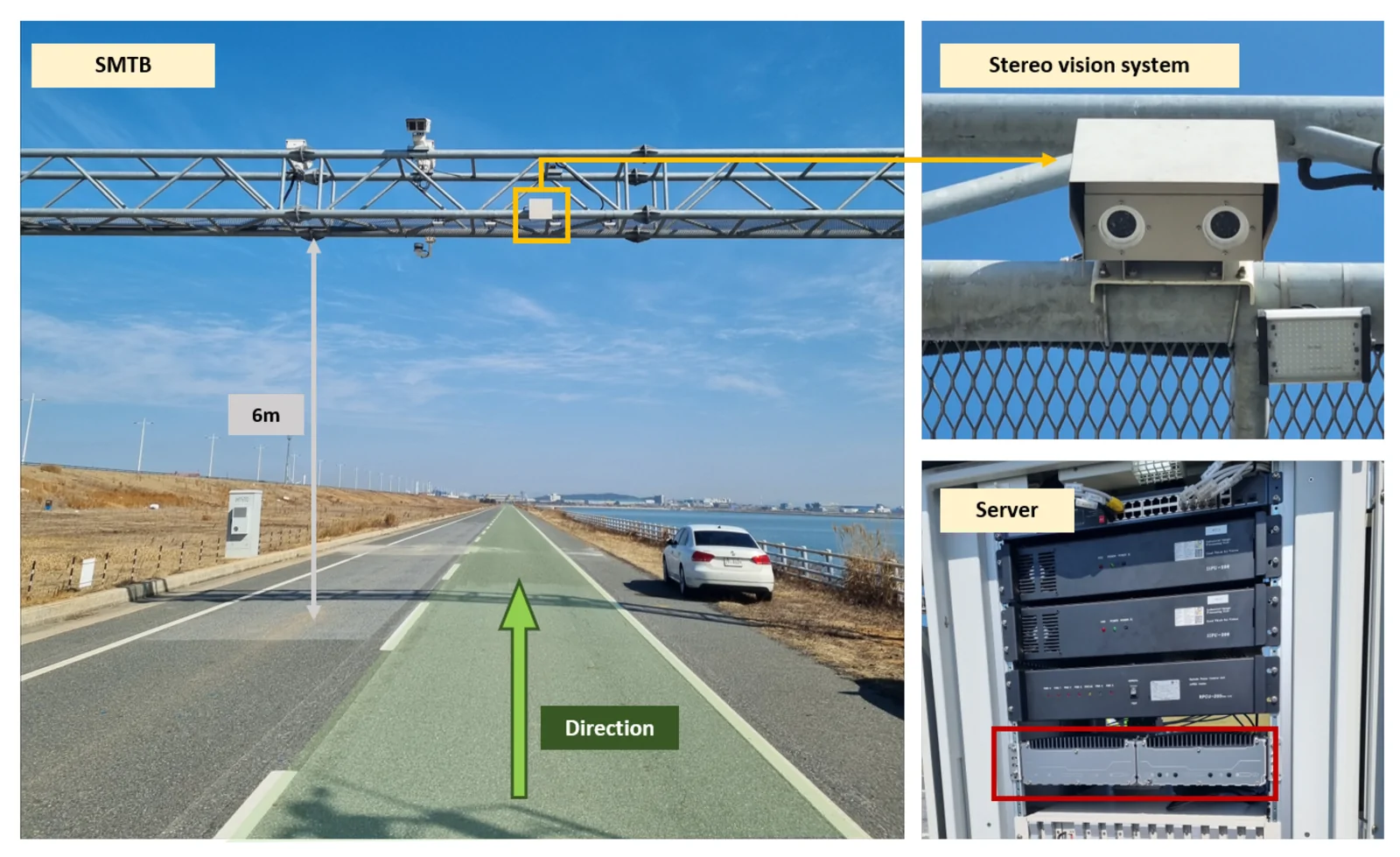
Installation of the stereo vision system and server at SMTB. The orange box indicates the stereo vision system, and the red box indicates the server.

To validate the proposed algorithm, a field environment was required in which a stereo vision system could measure a driving vehicle on the road. Figure 6 shows the field deployment of the stereo vision system on a roadway that vehicles do not normally drive, located at the Saemangeum Test Bed (SMTB) in Gunsan-si, Korea. The stereo vision system was installed on a pole at a height of 6 m in the direction perpendicular to the ground, so that the entire body of a driving cargo truck could be captured in the images. We used FLIR cameras (BFS-PGE-19S4C-C, Teledyne FLIR, Richmond, BC, Canada), which provide a resolution of 1616 × 1240 (2.0 MP) and a frame rate of 30 fps. A server (SEMIL-1341GC-NX, Neousys Technology Inc., Taipei, Taiwan) that runs the cargo-change detection program was installed inside an enclosure located 14 m from the pole.

### 4.2. Test Scenarios

**Figure 7:**
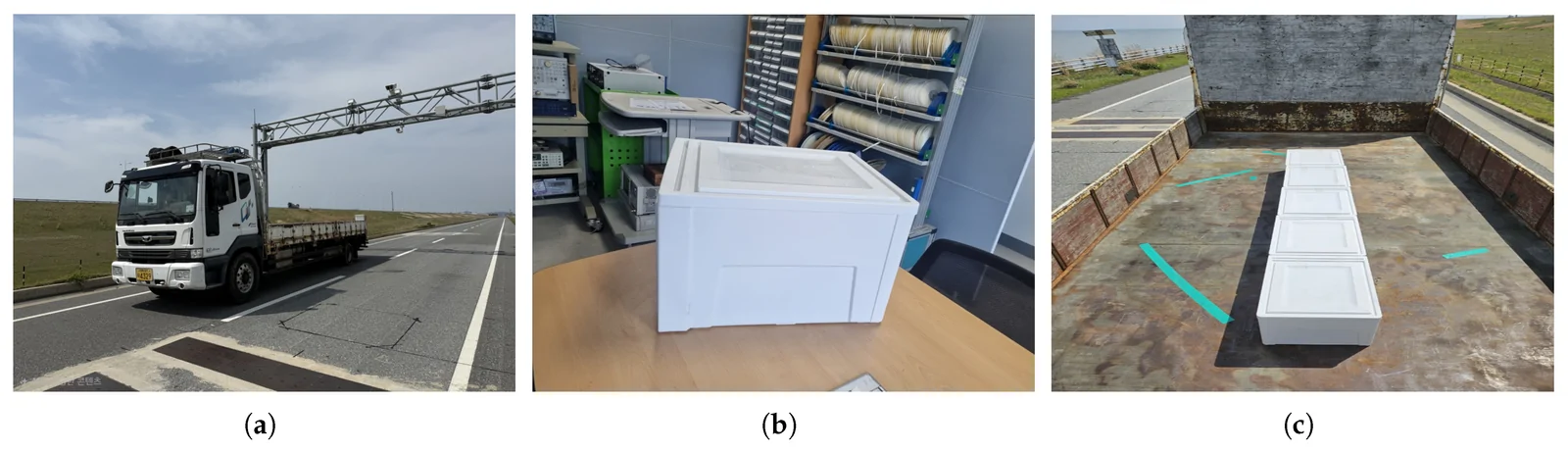
Type 1 test setup: (**a**) heavy-duty truck, (**b**) cargo box, and (**c**) cargo boxes loaded on the cargo bed.

In this section, we describe the two types of test scenarios designed to simulate changes in cargo load on the cargo bed of a truck: a controlled scenario (Type 1) and a realistic scenario (Type 2). In both scenarios, the truck performed repeated passes through the stereo vision system and the cargo load was modified between consecutive passes to generate load change events for detection. Figure 7 shows the vehicle and the cargo items used for the test drives in Type 1. A heavy-duty truck (Korea Three-Axle Industry Hanss, 6.7-ton payload capacity, manufacturer-classified as “heavy-duty”) was employed. The cargo items consisted of six identical, standardized boxes, each measuring 49 cm × 40 cm × 29 cm, and no other items were present on the cargo bed.

We conducted a total of 25 passes, yielding 24 load change events. Table 1 presents the full sequence of load configurations across all passes. The addition and removal phases were repeated twice: during the first addition phase (Pass No. 1–7), one box was added to the cargo bed per pass until all six boxes were loaded, and during the first removal phase (Pass No. 8–13), one box was removed per pass until the cargo bed was empty. The second addition phase (Pass No. 14–19) and the removal phase (Pass No. 20–25) followed the same procedure.

**Figure 8:**
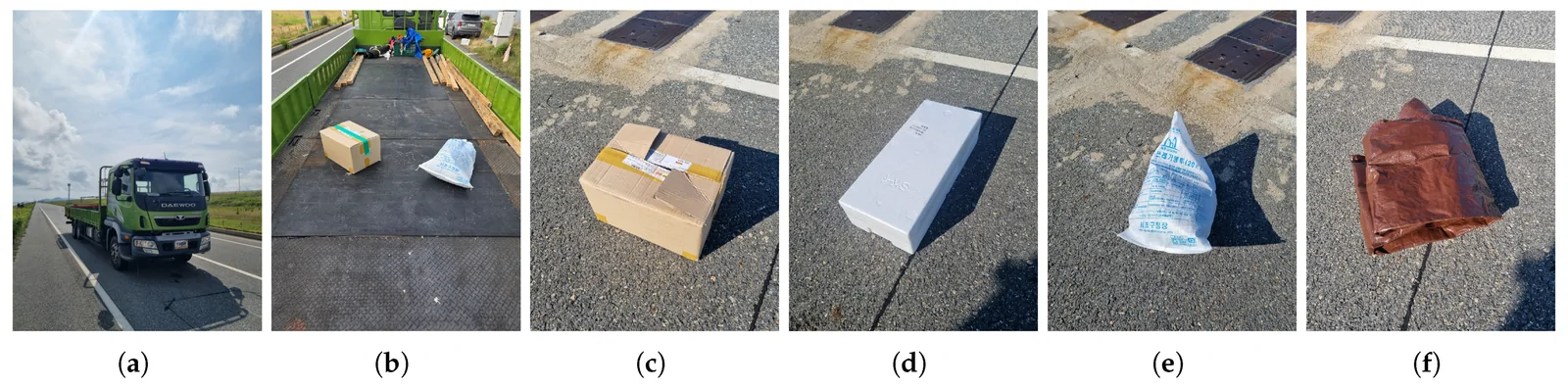
Type 2 test setup: (**a**) medium-duty truck, (**b**) cargo bed with other items, (**c**) cardboard box, (**d**) EPS block, (**e**) waste collection bag, and (**f**) tarpaulin.

In Type 2, a medium-duty truck (Tata Daewoo Prima Medium Cargo, 4.5-ton payload capacity, manufacturer-classified as “medium-duty”) was employed for the test drives. Figure 8 shows the vehicle and the four types of cargo items adopted in Type 2. Unlike Type 1, the cargo bed contained other items in addition to the test cargo, reflecting a more realistic loading condition. We selected four types of cargo items as representative examples of objects that frequently fall from vehicles on real roads: a cardboard box, an expanded polystyrene (EPS) block, a waste collection bag and a tarpaulin.

Table 2 presents the measured dimensions and surface material of the four cargo items used in the Type 2 test scenario. Among them, the waste collection bag has the smallest depth, at 100 mm, while the tarpaulin has the smallest width and length, at 270 mm and 300 mm, respectively. The surface materials range from the matte, textured cardboard box, EPS block, and polypropylene bag to the glossy, low-texture, waterproof-coated fabric of the tarpaulin.

We conducted a total of 24 passes, yielding 23 load change events. Table 3 presents the load configurations for each pass in Type 2. As shown in Table 3, the cargo items were placed on the cargo bed individually (Pass No. 2, 4, 6, 8) or in combination (Pass No. 10, 12, 14, 17, 19, 22), with empty and loaded states alternating across passes to produce a variety of load change conditions.

### 4.3. Evaluation Metrics

**Figure 9:**
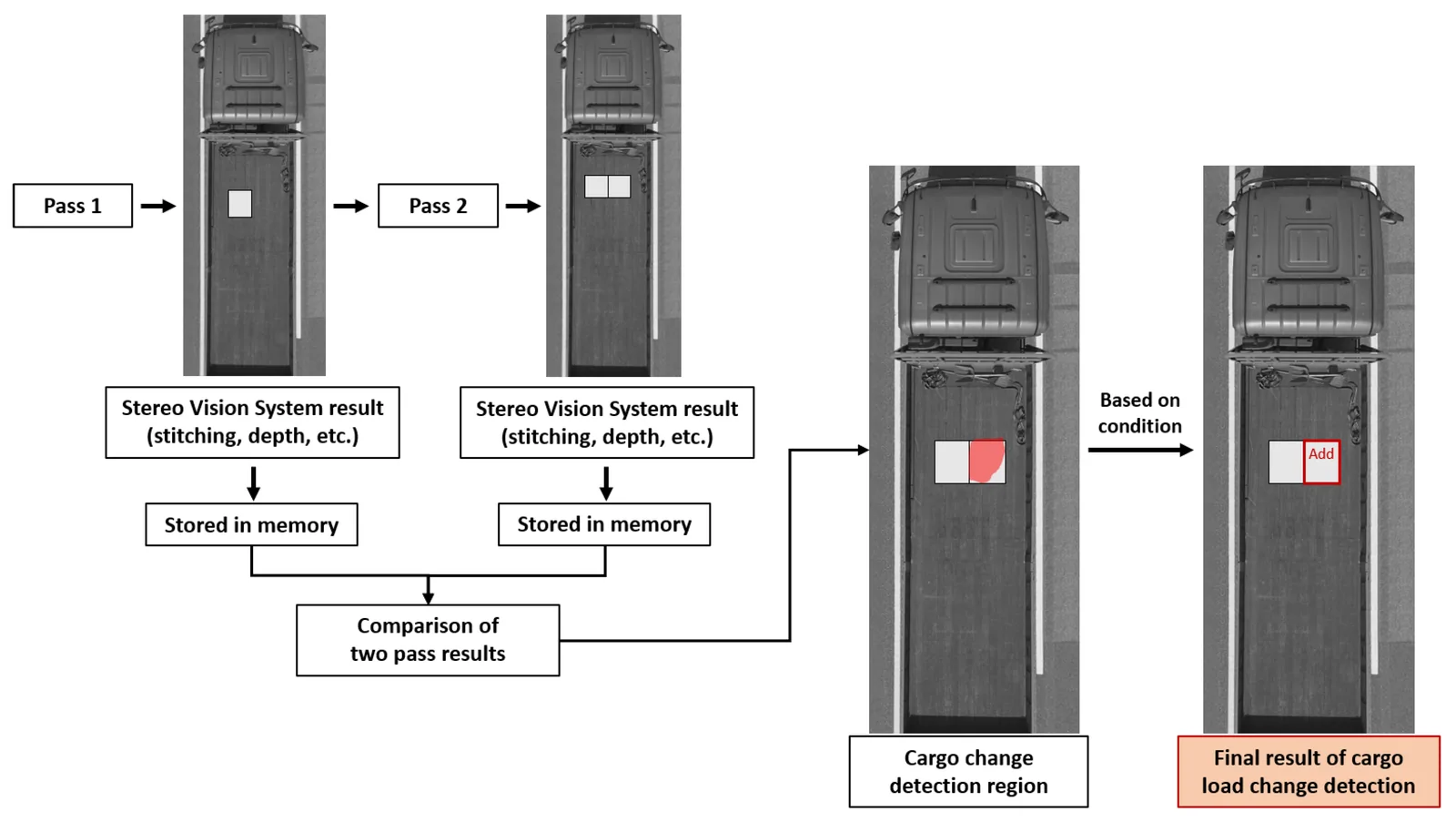
Overall process of cargo load change detection result output. The white region indicates the cargo item, and the red box indicates the detected addition.

Figure 9 illustrates the overall process of detecting cargo load changes. We perform two consecutive passes, a previous pass (Pass 1) and a current pass (Pass 2), using the cargo load change detection program. The stereo vision system result from each pass is stored in memory, and we compare the two pass results to detect any changes in the cargo load. Upon completion of the analysis, the final result of cargo load change detection is generated. When a cargo item is added, the corresponding region is labeled “Add” in the result. Conversely, if a cargo item is removed, the region is labeled “Remove.”

To quantitatively evaluate the detection performance, we define the cargo load change detection rate as follows:

(10) $$\mathrm{Detection}\;\mathrm{Rate}\;\left(\%\right)=\frac{\mathrm{Number}\;\mathrm{of}\;\mathrm{correctly}\;\mathrm{detected}\;\mathrm{load}\;\mathrm{change}\;\mathrm{events}}{\mathrm{Total}\;\mathrm{number}\;\mathrm{of}\;\mathrm{actual}\;\mathrm{load}\;\mathrm{change}\;\mathrm{events}}\times 100$$

We consider a detection result as correct only when the algorithm identifies both the type and location of the change in cargo load. In particular, we define two types of erroneous detection:

- Missed detection: the algorithm fails to detect any change in the cargo load.
- False detection: the algorithm incorrectly identifies a change in a region where no actual change has occurred.

Furthermore, we evaluate a result as incorrect if a correct detection and a false detection coexist in the same output, even if the actual changed region is identified. Only results that exclusively yield the correct change type and location, without missed or false detections, are counted as correct detections.

## 5. Results

In this section, we present the experimental results to validate the proposed cargo load change detection algorithm. We first evaluate the detection performance of the proposed algorithm for both Type 1 and Type 2 test scenarios, as described in Section 4.2, and then compare the results with those of previous methods to demonstrate the superiority of the proposed algorithm.

### 5.1. Evaluation of Cargo Load Change Detection

Table 4 reports the cargo load change detection results for the Type 1 and Type 2 test scenarios. In Type 1, the proposed algorithm achieved a detection rate of 100% (24/24), with no false or missed detections. On the other hand, in Type 2, the detection rate was 82.61% (19/23), with 7 false detections and no missed detections. Overall, the proposed algorithm achieved a detection rate of 91.49% (43/47) across both test scenarios.

**Figure 10:**
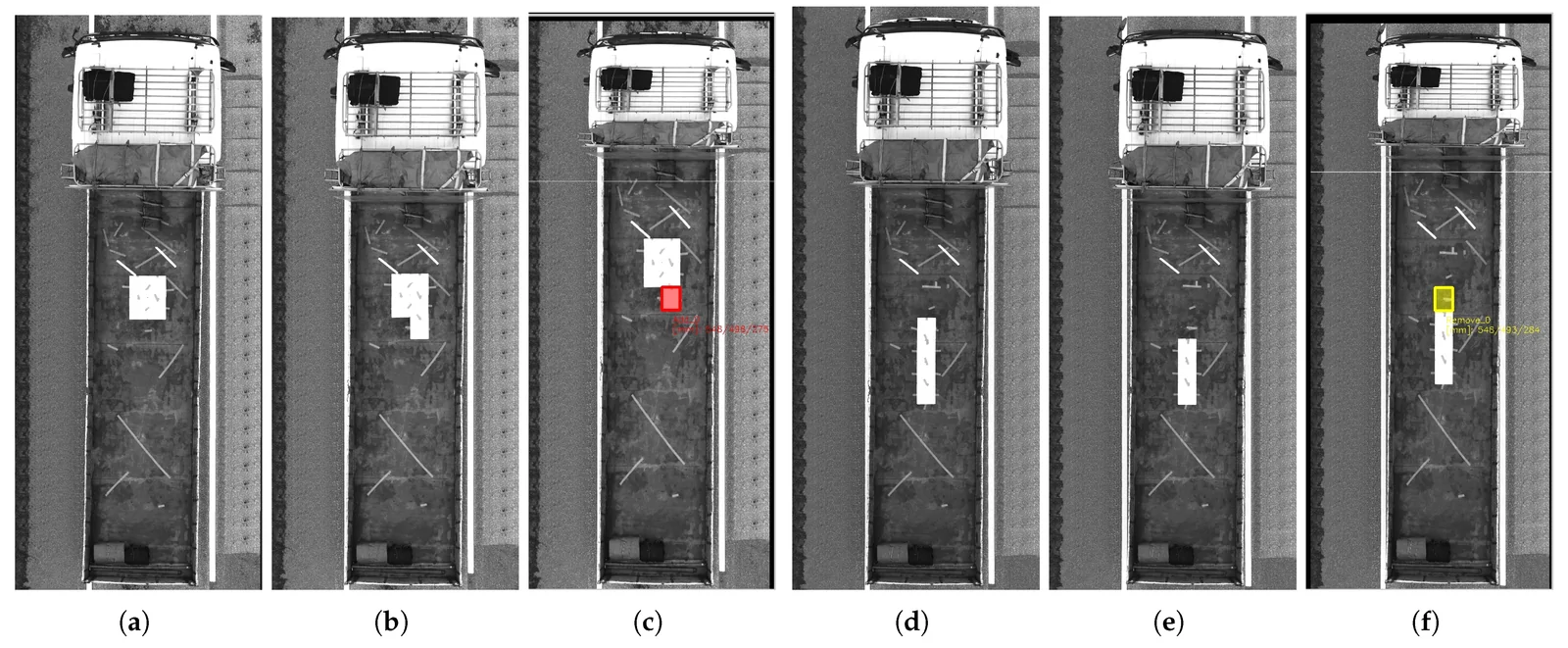
Examples of cargo load change detection results in Type 1: (**a**) Pass No. 5 (previous), (**b**) Pass No. 6 (current), (**c**) detection result showing the added cargo item marked with a red box, (**d**) Pass No. 21 (previous), (**e**) Pass No. 22 (current), and (**f**) detection result showing the removed cargo item marked with a yellow box.

In Type 1, the cargo items consisted of six identical boxes of uniform size and shape, which provided a simple and well-defined detection environment. Figure 10 presents examples of the detection results in Type 1. As shown in Figure 10a,b, a box was added to the cargo bed between Pass No. 5 and Pass No. 6, and Figure 10c shows that the proposed algorithm accurately detected the added region, marking it with a red box. Similarly, Figure 10d,e show a box removed from the cargo bed between Pass No. 21 and Pass No. 22, and Figure 10f confirms that the algorithm correctly identified the removed region, marking it with a yellow box.

Given the relatively large size of the boxes, the proposed algorithm was able to clearly distinguish the cargo change region from the surrounding area. Furthermore, we confirm that the algorithm accurately detected load changes even when the boxes were placed adjacent to one another on the cargo bed, which is a particularly encouraging result. From this result, it can be said that the proposed algorithm is robust to closely arranged cargo items under controlled conditions.

In the case of Type 2, the four different cargo items varied considerably in size, shape, and material, introducing greater complexity into the detection environment. Moreover, the cargo bed contained other items in addition to the test cargo, which further in-creased the difficulty of accurately detecting the load changes.

**Figure 11:**
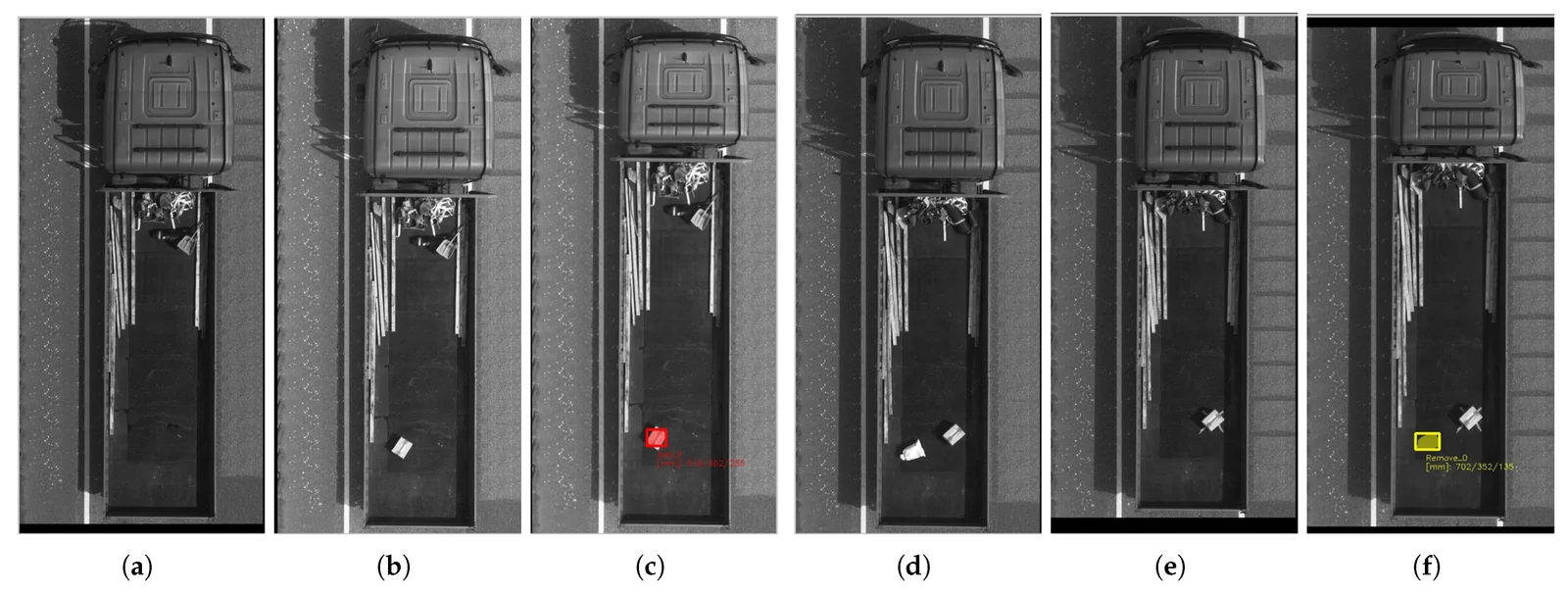
Examples of cargo load change detection results in Type 2: (**a**) Pass No. 1 (previous), (**b**) Pass No. 2 (current), (**c**) detection result showing the added cardboard box marked with a red box, (**d**) Pass No. 12 (previous), (**e**) Pass No. 13 (current), and (**f**) detection result showing the removed waste collection bag marked with a yellow box.

Figure 11 presents examples of the detection results in Type 2. As shown in Figure 11a,b, a cardboard box was added to the cargo bed between Pass No. 1 and Pass No. 2, and Figure 11c shows that the proposed algorithm accurately detected the added region, marking it with a red box. Similarly, Figure 11d,e show the waste collection bag removed from the cargo bed between Pass No. 12 and Pass No. 13, and Figure 11f confirms that the algorithm correctly identified the removed region, marking it with a yellow box. Nonetheless, we confirm that no missed detections were recorded, indicating that the proposed algorithm consistently identified the presence of a load change even under more complex conditions.

**Figure 12:**
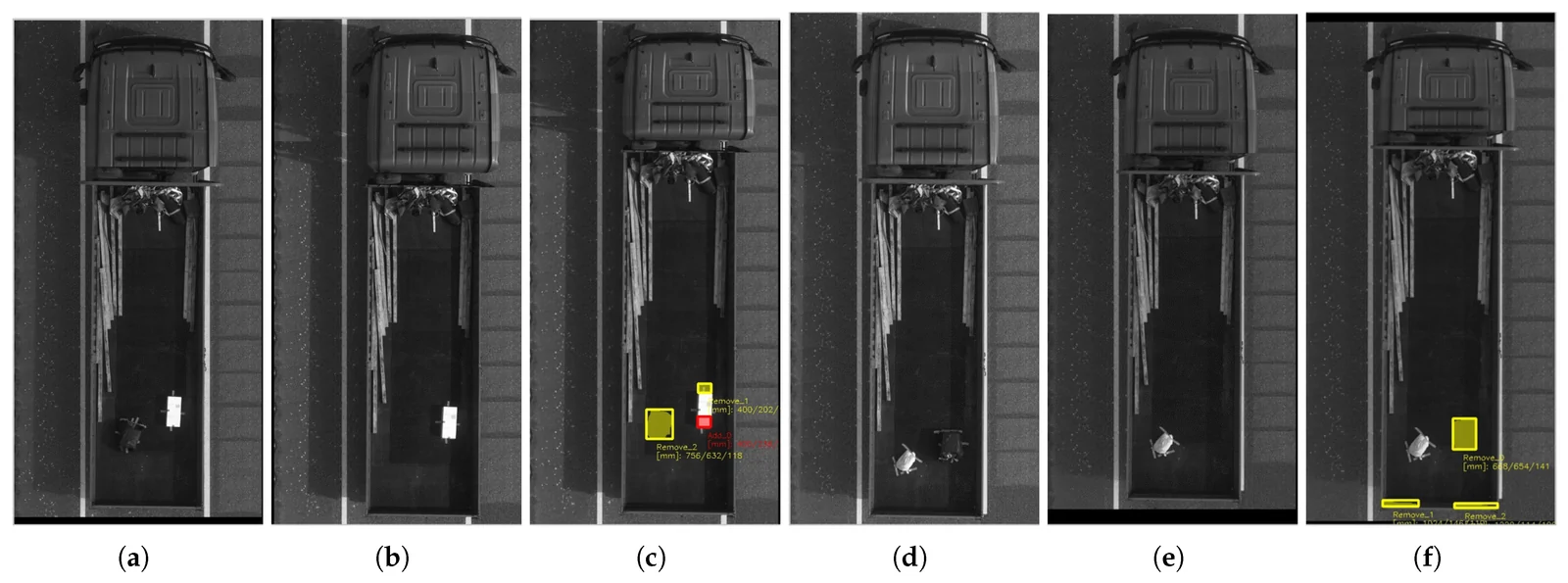
Examples of false detections in Type 2: (**a**) Pass No. 19 (previous), (**b**) Pass No. 20 (current), and (**c**) detection result showing the correctly detected removal of the tarpaulin, marked with a yellow box, together with two false detections near the unchanged EPS block; (**d**) Pass No. 22 (previous), (**e**) Pass No. 23 (current), and (**f**) detection result showing the correctly detected removal of the tarpaulin, together with false detections near the edge of the cargo bed.

To further illustrate the false detections observed in Type 2, Figure 12 presents two representative examples corresponding to the two error categories. As shown in Figure 12a,b, a tarpaulin was removed from the cargo bed between Pass No. 19 and Pass No. 20, while an EPS block remained unchanged in the same area. Figure 12c shows that the proposed algorithm correctly detected the removal of the tarpaulin, marking it with a yellow box. However, we observe that the algorithm additionally generated two false detections near the unchanged EPS block, incorrectly marking it as both removed and added. This result suggests that the proposed algorithm can confuse the front and back regions of an unchanged object, particularly under irregular placement conditions.

Figure 12d,e show a second example, in which a tarpaulin was removed from the cargo bed between Pass No. 22 and Pass No. 23, while a waste collection bag remained unchanged in the same area. Figure 12f shows that the proposed algorithm correctly detected the removal of the tarpaulin, marking it with a yellow box, but additionally generated false detections near the edge of the cargo bed, unrelated to either cargo item. This result indicates a residual alignment error rather than a misclassification of cargo appearance.

Table 5 presents a complete classification of the seven false detections observed in the Type 2 test scenario. Four cases (57.1%), occurring in Pass 18–19 and Pass 19–20, involve the EPS block and stem from the confusion between its front and back regions described above. The remaining three cases (42.9%), occurring in Pass 6–7 and Pass 22–23, are not associated with any cargo item and instead appear near the edge of the cargo bed, indicating a residual alignment error rather than a misclassification of cargo appearance.

To partially address the limited scale of the Type 2 evaluation, we additionally applied the proposed algorithm to 21 passes collected from other vehicle types and cargo configurations not included in Type 1 or Type 2. All 21 events were correctly detected, with no false or missed detections, corresponding to a 95% confidence interval of [83.9%, 100.0%].

Based on the results of the two test scenarios, Type 1 achieved a higher detection rate than Type 2, which we primarily attribute to the more uniform cargo size, shape, and placement in Type 1 compared to the diverse and irregularly placed cargo in Type 2. The remaining false detections arose from a residual alignment error near the edge of the cargo bed, unrelated to cargo type or placement. Nevertheless, the absence of missed detections in both scenarios demonstrates that the proposed algorithm reliably identifies cargo load changes regardless of the complexity of the loading conditions.

We note, however, that Type 1 and Type 2 were conducted using trucks of different classes (6.7-ton and 4.5-ton payload capacity, respectively), which may differ not only in cargo-bed dimensions but also in surface material, background texture, and geometric characteristics. These vehicle-specific differences may have also contributed to the observed gap in detection rate.

### 5.2. Comparison with Previous Methods

In this subsection, we evaluate the cargo load change detection performance obtained when applying different alignment algorithms for the Type 2 test scenario. To validate the effectiveness of the proposed alignment method, we compare it against a baseline without any alignment (None) and four widely used feature-based image alignment algorithms: SIFT, ORB, AKAZE, and XFeat.

As shown in Table 6, the proposed algorithm (Ours) attained the highest detection rate of 82.61% (19/23) among all compared methods, with 7 false detections and no missed detections. This result is meaningful, as missed detections pose a greater safety risk than false detections. Among the four conventional alignment algorithms, missed detections remained constant at 8 cases, whereas false detections varied considerably, ranging from 0 for SIFT to 14 for ORB. Specifically, SIFT and XFeat recorded a detection rate of 65.22% (15/23), followed by ORB and AKAZE at 60.87% (14/23). The baseline without alignment (None, 2D image) yielded the lowest detection rate of 26.09% (6/23), with 55 false detections and 8 missed detections.

To assess the contribution of the depth modality separately from that of the proposed one-dimensional projection matching, we omitted the alignment step and used depth information instead of a two-dimensional image. None (Depth) achieved a detection rate of 43.48% (10/23), with 23 false detections and no missed detections. This result shows that depth-based comparison alone outperforms None (2D image) and avoids all eight missed detections observed across every 2D-image baseline.

In addition, comparing None (Depth) to the proposed algorithm isolates the contribution of the proposed one-dimensional projection matching while holding the depth modality constant: introducing this step nearly doubles the detection rate and reduces false detections from 23 to 7. Overall, these results indicate that both the depth-based modality and the proposed alignment strategy contribute independently to the performance of the proposed algorithm.

**Figure 13:**
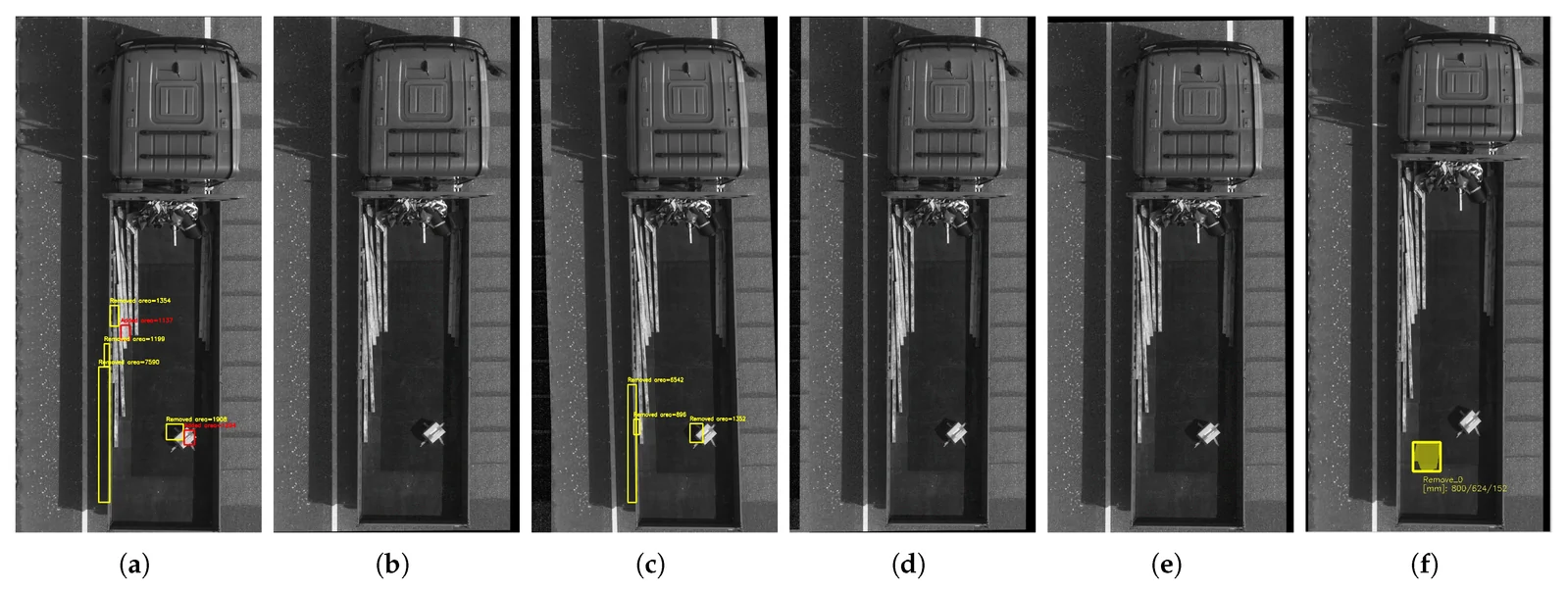
Detection results for the same tarpaulin-removal event (Pass No. 14 to Pass No. 15) in Type 2: (**a**) None, (**b**) SIFT, (**c**) ORB, (**d**) AKAZE, (**e**) XFeat and (**f**) the proposed algorithm (Ours), correctly detecting the removed region and marking it with a yellow box.

This dependence on two-dimensional image information is also evident in the missed detections: all five 2D-image baselines consistently recorded eight missed detections, all of which involved the tarpaulin (Figure 8f). Figure 13 presents a representative example for the same pass pair (Pass No. 14 to Pass No. 15), showing that none of the five 2D-image baselines correctly detected the tarpaulin removal. In contrast, the proposed algorithm accurately identified the removed region, marking it with a yellow box.

## 6. Discussion

Section 5.2 shows that missed detections remained constant at 8 cases across the four conventional alignment algorithms in the Type 2 test scenario, while false detections varied considerably. In this section, we discuss the cause of this pattern, examine the robustness and limitations of the proposed algorithm under challenging surface and height conditions, compare the processing time across algorithms, and summarize the advantages of the proposed algorithm.

### 6.1. Robustness to Surface Conditions

We observe that all 8 missed detections recorded by each conventional algorithm correspond to the same load change events, each involving the tarpaulin shown in Figure 8f. This consistency indicates a shared cause rather than a limitation specific to any single algorithm, namely the close color and texture similarity between the tarpaulin and the surrounding cargo bed, which limits the effectiveness of two-dimensional color-based features [43].

**Figure 14:**
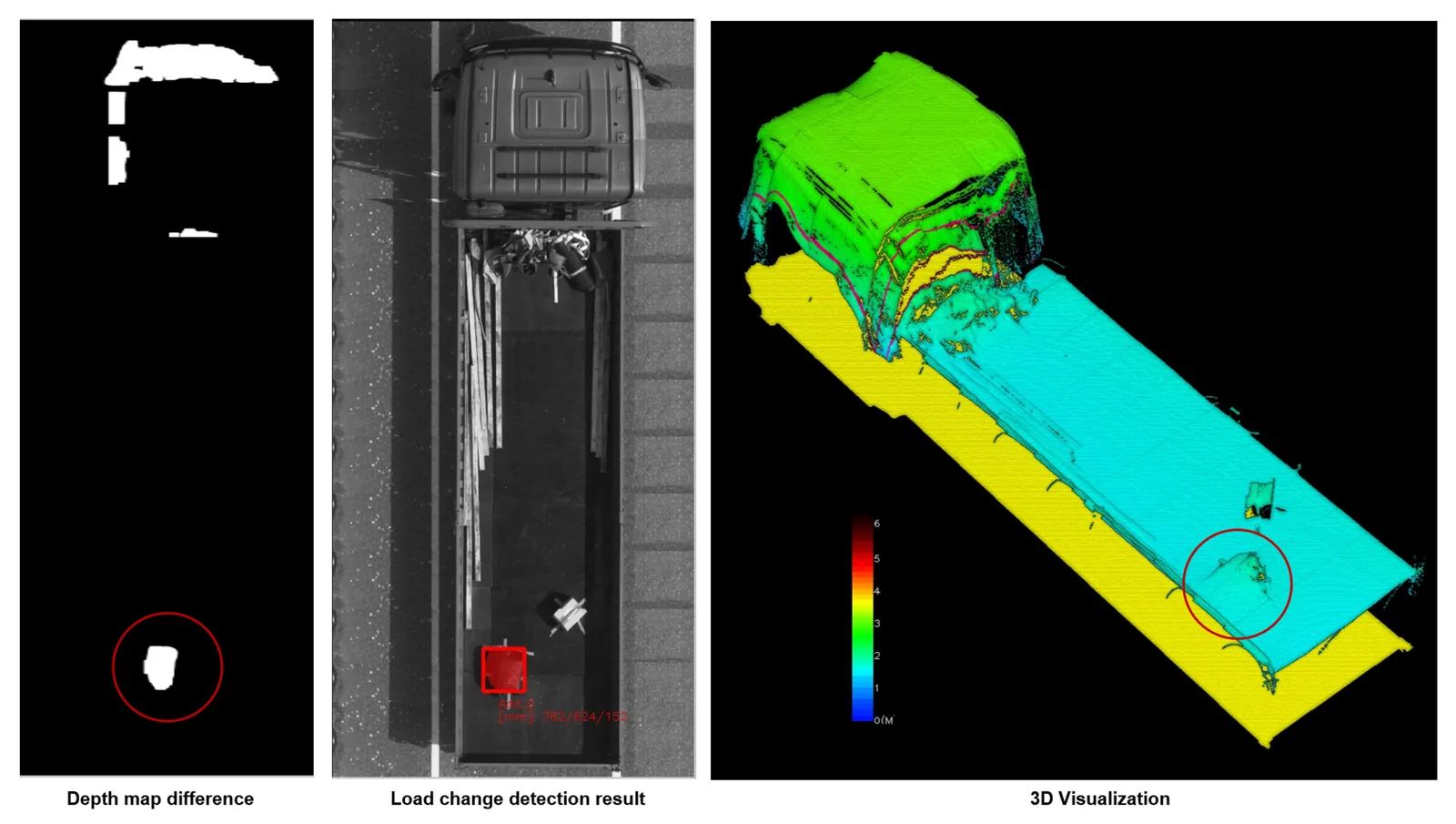
Result of tarpaulin addition detection obtained by the proposed algorithm. The red circle indicates the tarpaulin region, and the red box indicates the detected addition.

Figure 14 presents the corresponding detection result obtained by the proposed algorithm for the same tarpaulin addition event, which utilizes depth information instead of color or texture. With the tarpaulin region marked by a red circle, we confirm that both the depth map difference and the three-dimensional visualization clearly capture this region, and it is correctly detected as a load change, marked with a red box in the detection result. This result demonstrates that the proposed algorithm remains unaffected by the color and texture similarity that causes the conventional algorithms to miss this type of event.

**Figure 15:**
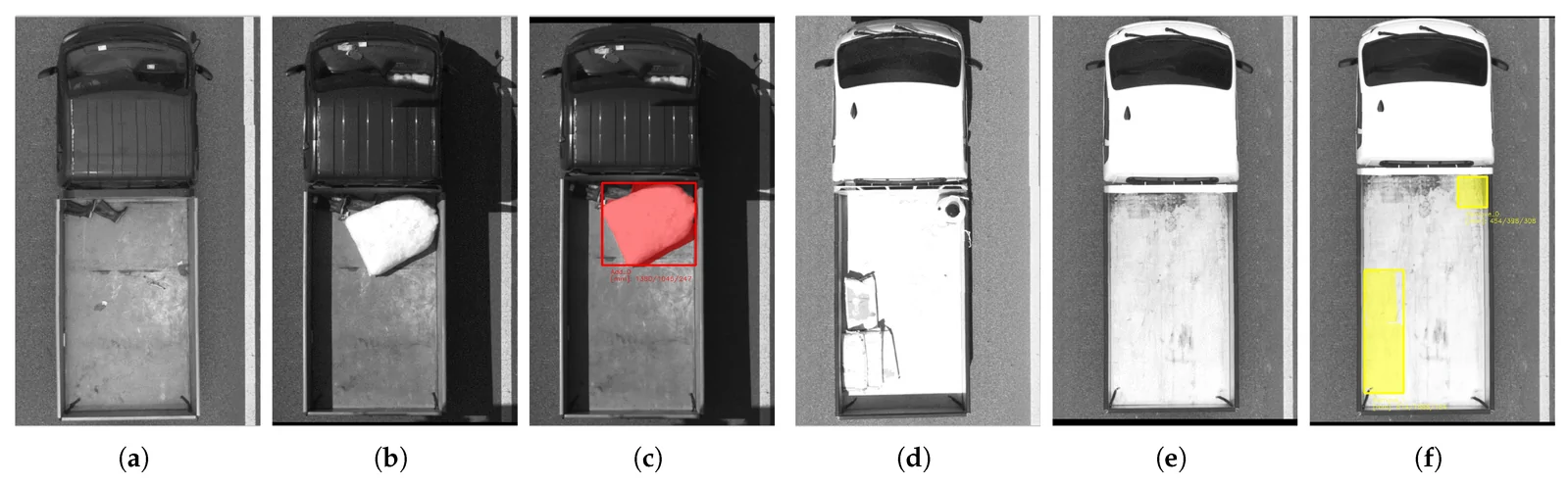
Examples of correct detection under challenging surface conditions in field operation: (**a**) previous pass, (**b**) current pass, (**c**) detection result; (**d**) previous pass, (**e**) current pass, (**f**) detection result.

The proposed algorithm has also demonstrated tolerance to reflective, low-texture surfaces in field operation. Figure 15c shows a plastic bag with a smooth, low-texture surface, correctly detected as an added load, while Figure 15f presents removed loads correctly detected despite strong sunlight reflection on the cargo-bed surface. These results indicate that the proposed algorithm maintains a degree of robustness under conditions involving high reflectivity or low texture.

These observations, however, should be interpreted within the scope of the conditions examined in this study. The experiments were conducted over a single day, from 09:00 to 16:00, under natural daylight without any auxiliary lighting, and the current dataset does not include scenarios involving substantial variation in ambient lighting, such as low-light or nighttime operation, nor cargo surfaces with texture lower than that of the tested cases, such as transparent materials. Under such circumstances, stereo matching may become less reliable, degrading the accuracy of the resulting depth map. Complementing depth information with color or texture cues could help distinguish cargo in such conditions; this approach would nonetheless be of limited value for cargo such as the tarpaulin discussed above, whose color and texture closely resemble those of the cargo bed.

### 6.2. Measurement Resolution

The candidate-region extraction step applies a minimum height threshold to suppress noise, so that height differences below this threshold are excluded from consideration regardless of their actual presence. As a result, cargo with a height difference nearly indistinguishable from the cargo-bed floor, such as a flattened object with negligible relief, remains undetected.

Regarding the measurement resolution of the proposed system, we estimate the depth, width, and length resolution from the stereo geometry and the pixel size of the camera. Table 7 shows the resulting resolution along all three axes across the range of distances involved in the experimental setup, based on the baseline of 175 mm and the focal length of 6 mm used in this study. The depth resolution assumes a disparity precision of one pixel, while the width and length resolution maps to the physical extent covered by a single pixel. At 5 m, close to the distance from the camera to the cargo bed, the depth resolution is approximately 107.1 mm, and the width and length resolution is approximately 3.75 mm/px.

These estimates are consistent with the empirical results in Table 2. The smallest detected depth, 100 mm for the waste collection bag, is close to the estimated value at 5 m, indicating that the effective depth resolution of the proposed system is consistent with a single-pixel disparity precision. Through the Type 2 experiments, we confirm that the proposed system reliably detects load changes with a depth of at least 100 mm, a width of at least 270 mm, and a length of at least 300 mm, evaluated based on the resulting geometric footprint on the depth map rather than weight.

### 6.3. Processing Time Comparison

Table 8 reports the processing time of each alignment algorithm for the Type 1 and Type 2 test scenarios. We execute all algorithms, including XFeat, on the CPU to ensure a fair comparison of processing time, as the algorithms other than XFeat do not utilize GPU acceleration. Based on the average processing time, ORB shows the best performance at 212.9 ms, followed closely by the proposed algorithm (Ours) at 221.3 ms. Notably, Ours records a substantially shorter processing time than SIFT, AKAZE, and XFeat, despite relying on three-dimensional depth information rather than two-dimensional color features. XFeat shows the worst performance at 649.7 ms, considerably higher than the other conventional algorithms. This suggests that the substantially higher processing time of XFeat is attributable to this CPU execution rather than to an inherent limitation of the algorithm itself.

We therefore conclude that the proposed algorithm achieves a favorable balance between detection accuracy and processing time that the conventional algorithms fail to provide. This advantage stems from its depth-based nature: unlike the conventional algorithms, which rely on two-dimensional color and texture information, the proposed algorithm is less dependent on such similarity between cargo items and the cargo bed. It also avoids the heavy computational cost associated with hand-crafted or learning-based feature extraction. These results demonstrate that the proposed algorithm is well suited for the accurate and real-time detection of cargo load changes in practical field conditions.

## 7. Conclusions

In this study, we propose a method for detecting cargo load changes on trucks that combines depth map normalization and one-dimensional projection matching applied to data acquired through a stereo vision system, accurately aligning cargo-bed images captured at different passes. Existing cargo inspection practices rely primarily on manual visual inspection, which requires substantial manpower, or weight-based systems such as WIM sensors, which fail to reliably detect load changes involving items of similar weight. To address these limitations, we developed a system that directly captures and compares the actual loading state of cargo using depth information, which is inherently independent of the color and texture of the captured objects.

We evaluate the proposed method through field experiments at SMTB, in terms of detection performance across two test scenarios with different cargo item types and placement conditions, and comparison with conventional alignment algorithms, including SIFT, ORB, AKAZE, and XFeat. The proposed algorithm achieves an overall detection rate of 91.49% (43/47) across both scenarios, considerably outperforming all conventional algorithms while maintaining a processing time close to that of ORB, the fastest among the compared methods. In particular, we identify that the missed detections consistently observed among the conventional algorithms stem from the color and texture similarity between certain cargo items and the cargo bed, a limitation that the proposed algorithm overcomes owing to its depth-based nature.

We believe that this study can facilitate future research on cargo load change detection for trucks. Potential future work includes reducing the false detections observed under irregular cargo placement conditions, particularly the confusion between the front and back regions of an unchanged object. In addition, conducting a controlled experiment using the same vehicle across different cargo configurations would enable a more detailed analysis of the factors underlying the difference in detection rate observed between the two test scenarios. Furthermore, extending the evaluation to a wider range of truck types, cargo configurations, illumination conditions, and surface materials with varying texture and reflectivity would help validate the robustness of the proposed algorithm in more diverse field environments.

## Figures and Tables

**Table 1:** Load configuration sequence for Type 1 test scenario.

Pass No.	Phase	No. of Boxes in Cargo Bed	Load Change
1–7	Addition (1st)	0 → 6	Addition
8–13	Removal (1st)	6 → 0	Removal
14–19	Addition (2nd)	0 → 6	Addition
20–25	Removal (2nd)	6 → 0	Removal

**Table 2:** Measured dimensions and surface material of the cargo items used in the Type 2 test scenario.

Cargo Item	Depth (mm)	Width (mm)	Length (mm)	Surface Material
Cardboard box	240	320	350	Corrugated cardboard (matte, textured)
EPS block	190	300	690	Expanded polystyrene (matte, finely textured)
Waste collection bag	100	300	500	Polypropylene (matte, textured)
Tarpaulin	120	270	300	Waterproof-coated fabric (glossy, low-texture)

**Table 3:** Load configuration sequence for Type 2 test scenario. The cargo item labels (c)–(f) correspond to those shown in Figure 8.

Pass No.	Cargo Items in Cargo Bed	Load Change
1	Empty	-
2	(c)	Addition
3	Empty	Removal
4	(d)	Addition
5	Empty	Removal
6	(e)	Addition
7	Empty	Removal
8	(f)	Addition
9	Empty	Removal
10	(c), (d)	Addition
11	(c)	Removal
12	(c), (e)	Addition
13	(c)	Removal
14	(c), (f)	Addition
15	(c)	Removal
16	Empty	Removal
17	(d), (e)	Addition
18	(d)	Removal
19	(d), (f)	Addition
20	(d)	Removal
21	Empty	Removal
22	(e), (f)	Addition
23	(e)	Removal
24	Empty	Removal

**Table 4:** Cargo load change detection results for the Type 1 and Type 2 test scenarios.

Test Scenario	# of False Detection	# of Missed Detection	Detection Rate
Type 1	0	0	100% (24/24)
Type 2	7	0	82.61% (19/23)
**Total**	7	0	**91.49%** (43/47)

**Table 5:** Classification of the seven false detections observed in the Type 2.

No.	Pass Pair	Cargo Item	Error Category
1	6, 7	-	Residual alignment error
2	18, 19	EPS block	Visually/geometrically similar regions
3	18, 19	EPS block	Visually/geometrically similar regions
4	19, 20	EPS block	Visually/geometrically similar regions
5	19, 20	EPS block	Visually/geometrically similar regions
6	22, 23	-	Residual alignment error
7	22, 23	-	Residual alignment error

**Table 6:** Comparison of cargo load change detection performance across different alignment algorithms for the Type 2 test scenario.

Alignment Algorithm	Modality	# of False Detection	# of Missed Detection	Detection Rate
None	2D image	55	8	26.09% (6/23)
SIFT	2D image	0	8	65.22% (15/23)
ORB	2D image	14	8	60.87% (14/23)
AKAZE	2D image	2	8	60.87% (14/23)
XFeat	2D image	1	8	65.22% (15/23)
None	Depth	23	0	43.48% (10/23)
**Ours**	Depth	7	0	**82.61%** (19/23)

**Table 7:** Theoretical measurement resolution at varying distances from the camera to the cargo bed.

Distance (m)	Depth Resolution (mm)	Width/Length Resolution (mm/px)
1	4.3	0.75
2	17.1	1.50
3	38.6	2.25
4	68.6	3.00
5	107.1	3.75
6	154.3	4.50

**Table 8:** Comparison of processing time across different alignment algorithms for the Type 1 and Type 2 test scenarios.

Alignment Algorithm	Type 1 (ms)	Type 2 (ms)	Average (ms)
None	74.5	51.3	62.9
SIFT	451.7	345.5	398.6
ORB	219.5	206.2	212.9
AKAZE	334.9	269.7	302.3
XFeat	654.9	644.5	649.7
Ours	235.7	206.8	221.3

## Data Availability

The raw data supporting the conclusions of this article will be made available by the authors on request.

## Abbreviations

The following abbreviations are used in this manuscript:

SIFT	Scale-Invariant Feature Transform
ORB	Oriented FAST and Rotated BRIEF
AKAZE	Accelerated-KAZE
WIM	Weigh-in-motion
SMTB	Saemangeum Test Bed
EPS	Expanded polystyrene
